# Wearable Technology May Assist in Retraining Foot Strike Patterns in Previously Injured Military Service Members: A Prospective Case Series

**DOI:** 10.3389/fspor.2021.630937

**Published:** 2021-02-26

**Authors:** Donald L. Goss, Daniel J. Watson, Erin M. Miller, Amy N. Weart, Eliza B. Szymanek, Gregory M. Freisinger

**Affiliations:** ^1^Department of Physical Therapy, High Point University, High Point, NC, United States; ^2^15th Medical Group, Joint Base Pearl Harbor—Hickam, Honolulu, HI, United States; ^3^Baylor University—Keller Army Community Hospital Division 1 Sports Physical Therapy Fellowship, West Point, NY, United States; ^4^Department of Physical Therapy, Keller Army Community Hospital, West Point, NY, United States; ^5^Madigan Army Medical Center, Tacoma, WA, United States; ^6^Department of Civil and Mechanical Engineering, United States Military Academy, West Point, NY, United States

**Keywords:** wearable technology, running biomechanics, loading rate, cadence, foot strike, gait-retraining

## Abstract

A rearfoot strike (RFS) pattern with increased average vertical loading rates (AVLR) while running has been associated with injury. This study evaluated the ability of an instrumented sock, which provides real-time foot strike and cadence audio biofeedback, to transition previously injured military service members from a RFS to a non-rearfoot strike (NRFS) running pattern. Nineteen RFS runners (10 males, 9 females) were instructed to wear the instrumented socks to facilitate a change in foot strike while completing an independent walk-to-run progression and lower extremity exercise program. Kinetic data were collected during treadmill running while foot strike was determined using video analysis at initial (T1), post-intervention (T2), and follow-up (T3) data collections. Nearly all runners (18/19) transitioned to a NRFS pattern following intervention (8 ± 2.4 weeks after the initial visit). Most participants (16/18) maintained the transition at follow-up (5 ± 0.8 weeks after the post-intervention visit). AVLR of the involved and uninvolved limb decreased 29% from initial [54.7 ± 13.2 bodyweights *per sec* (BW/s) and 55.1 ± 12.7 BW/s] to post-intervention (38.7 ± 10.1 BW/s and 38.9 ± 10.0 BW/s), respectively. This effect persisted 5-weeks later at follow-up, representing an overall 30% reduction on the involved limb and 24% reduction on the uninvolved limb. Cadence increased from the initial to the post-intervention time-point (*p* = 0.045); however, this effect did not persist at follow-up (*p* = 0.08). With technology provided feedback from instrumented socks, approximately 90% of participants transitioned to a NRFS pattern, decreased AVLR, reduced stance time and maintained these running adaptations 5-weeks later.

## Introduction

Running is a popular form of exercise among all military service branches, and it is associated with a high rate of injuries (van Gent et al., 2007; Hauret et al., 2015; Molloy, 2016). Individual kinetic (force) and kinematic (movement) variations in running biomechanics have been proposed as potential risk factors for injury (Ceyssens et al., 2019). Foot strike pattern (FSP) is a kinematic component of running defined by the position of the foot at the point of initial contact with the ground. Approximately 80% of shod runners, those who run with a shoe on, use a rearfoot strike (RFS) pattern (Hasegawa et al., 2007; Lieberman et al., 2010; Almeida et al., 2015). FSPs have been suggested to affect important loading biomechanical variables related to running injury risk to include average vertical loading rate (AVLR), the rate at which force is applied to the body during loading response. It is well documented that AVLRs are greater in individuals who run with a RFS pattern versus a non-rearfoot strike pattern (NRFS) (Lieberman et al., 2010; Zadpoor and Nikooyan, 2011; Goss and Gross, 2013; Almeida et al., 2015; Goss et al., 2015; Miller et al., 2020; Xu et al., 2020). Of note, AVLRs greater than 70 BW/s have been associated with tibial and metatarsal stress fractures (Zifchock et al., 2006; Zadpoor and Nikooyan, 2011), patellofemoral pain syndrome (Johnson et al., 2020), and plantar fasciitis (Pohl et al., 2009). It is plausible that altering running biomechanics to reduce AVLR, and thereby improving shock attenuation, may be beneficial in the treatment and prevention of injuries (van der Worp et al., 2016).

Altering FSP from RFS to NRFS is a gait retraining technique commonly employed by clinicians to prevent and manage injuries (Barton et al., 2016; Roper et al., 2016; Miller et al., 2020). Reductions in AVLR, peak vertical ground reaction force (vGRF) (greatest magnitude of force during one step), and stance time have been achieved in healthy (Huang et al., 2019) and previously injured runners (Diebal et al., 2012; Roper et al., 2016; Miller et al., 2020) after transitioning from a RFS to a NRFS running pattern. Reductions in pain and improvements in function have also been reported following NRFS gait retraining (Roper et al., 2016; Miller et al., 2020). The ideal feedback method, technology, and length of intervention needed to change a person's FSP to achieve these outcomes is unknown and further research is required to address the efficacy and effectiveness of different feedback mechanisms (Tate and Milner, 2010).

Previous reports have shown the effectiveness of real-time biofeedback for gait retraining in healthy and previously injured runners (Cheung and Davis, 2011; Crowell and Davis, 2011; Noehren et al., 2011; Diebal et al., 2012; Willy et al., 2016) with changes maintained at a 1-month follow-up (Crowell and Davis, 2011; Willy et al., 2016). Visual, auditory, and tactile real-time feedback are common techniques used for gait retraining (Vannatta and Kernozek, 2015; Warne et al., 2017; Miller et al., 2020); however, visual feedback may lack real-world utility (Van Hooren et al., 2020) limiting runners to watching a display. Wearable technology with auditory feedback has been reported to successfully manipulate gait technique (Van Hooren et al., 2020) including transitioning FSP in healthy runners (Phanpho et al., 2019; Chan et al., 2020a). However, more research is needed to understand the usefulness and effectiveness of wearable technology with audio biofeedback to transition FSP in previously injured runners (DeJong and Hertel, 2018).

Wearable technology was recently developed which consists of an instrumented sock that is Bluetooth enabled and can provide runners with real-time biofeedback. When this device is paired with a smart device, the user can receive real-time auditory and/or visual biofeedback regarding FSP, cadence, running pace, total distance covered, elevation changes, and stance time. A change in foot strike pattern from RFS to NRFS has been observed in healthy runners when utilizing visual feedback from the instrumented socks (Phanpho et al., 2019). However, there are inherent limitations with visual feedback mechanisms and the gait changes the authors described were only observed acutely. Using other forms of real-time biofeedback with the instrumented sock and longitudinal follow-up to better understand gait retraining retention would be beneficial to explore.

The purpose of this case series was to examine the effectiveness of the instrumented sock to transition previously injured runners to a NRFS running pattern using real-time audio biofeedback at similar time points to previous studies (Cheung and Davis, 2011; Crowell and Davis, 2011; Noehren et al., 2011; Diebal et al., 2012; Willy et al., 2016). Secondarily, we examined pain scores, functional outcome measures, AVLR, cadence, peak vGRF, and stance time throughout the training protocol. We hypothesized the instrumented socks would enable approximately 50% of previously injured runners to transition from a RFS to a NRFS running pattern (Morris et al., 2020). Additionally, we hypothesized that the runners who transitioned to a NRFS pattern would exhibit no increase in pain, increased functional outcome scores, decreased AVLR, increased cadence, and reduced stance time (Miller et al., 2020).

## Methods

### Study Design

This was a prospective case series study involving patients recovering from lower extremity injury or surgery. A one-way repeated measures design with three timepoints was utilized (initial, post-intervention, and follow-up) to examine the effectiveness of the instrumented sock to transition patients to a NRFS running pattern. The initial visit (T1) took place at the time of enrollment. After the initial visit, the NRFS intervention was implemented. The post-intervention timepoint (T2) occurred between 6 and 10 weeks after the initial visit to assess the short-term effectiveness of the NRFS intervention. Finally, the follow-up timepoint (T3) took place ~4 weeks after the post-intervention visit to assess retention of the NRFS intervention. The primary outcome for this study was a NRFS running pattern and the secondary outcome of interest was AVLR. Based on an *a priori* power analysis (G^*^Power, version 3.1.9.2, Heinrich-Heine-Universitat Dusseldorf, Dusseldorf, Germany) of previously published data (Goss et al., 2015; Morris et al., 2020) using AVLR and cadence for a large effect size (*Cohen's d* = 0.80) with α set at 0.05, we determined at least nine participants were required for adequate power. To account for an above average attrition and possible injury or re-injury rate, we aimed to enroll a total of 23 runners.

### Participants

Inclusion criteria included individuals between 18 and 60 years of age who were a Department of Defense beneficiary (Active Duty Soldier, Cadet, or military dependent), owned a smart device, had a history of a lower extremity injury or surgery in the 12 months prior to study enrollment, and were cleared to return to running by their medical provider (primary care physician, orthopedic surgeon, or physical therapist). Exclusion criteria included low back pain or vestibular dysfunction in the previous 3 months, any history of a forefoot or midfoot fracture, current NRFS running pattern (following video analysis as described below), or self-reported inability to walk two miles pain-free. The study protocol was approved by the Keller Army Community Hospital Institutional Review Board, and written consent was obtained prior to participation.

**Figure 1:**
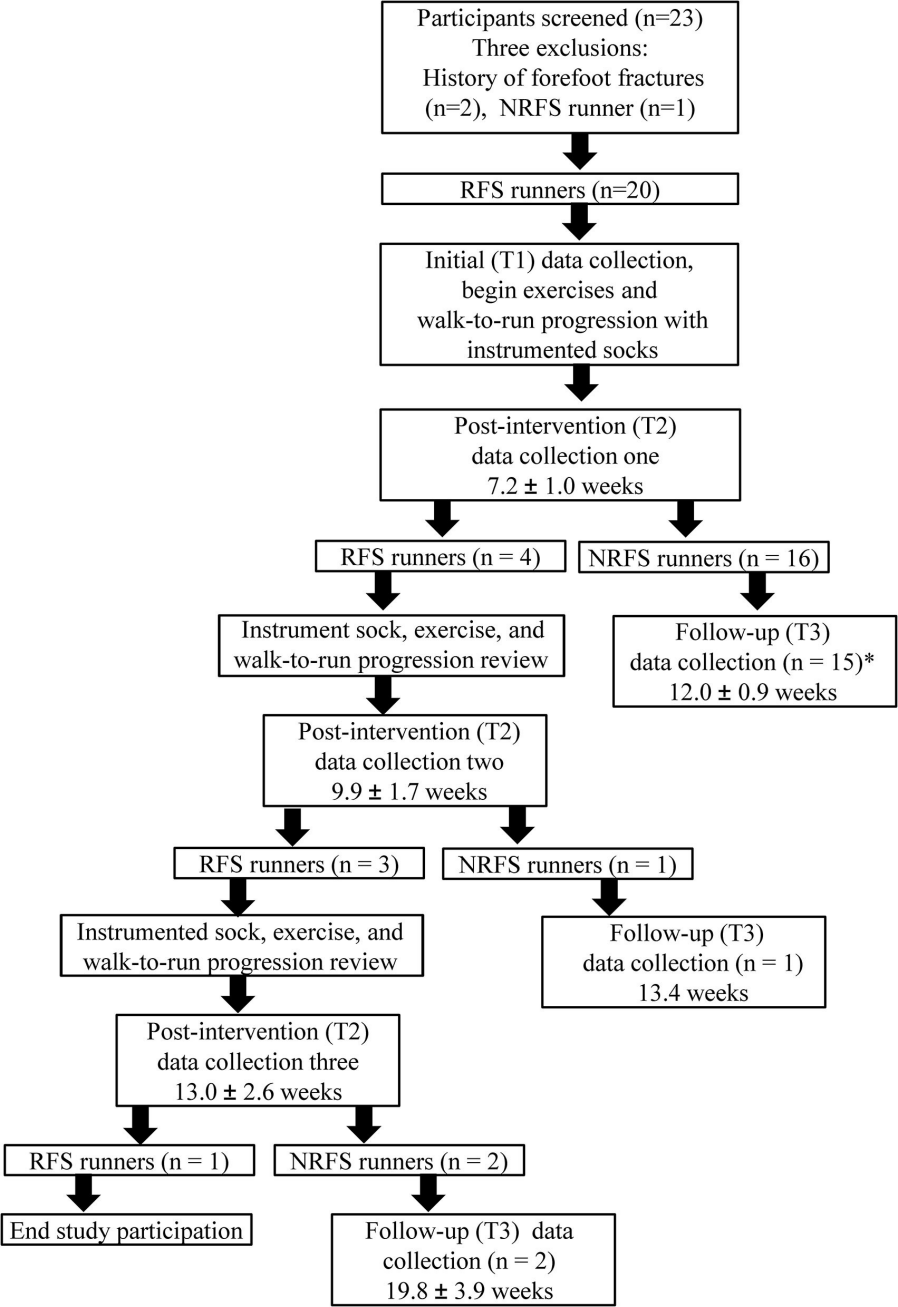
Participant flowchart and study methodology. RFS, rearfoot strike; NRFS, non-rearfoot strike. Values are reported as mean and standard deviation from the day 0 point of the study. *One participant was diagnosed with mononucleosis prior to the follow-up data collection. To prevent bias with an intention to treat analysis, all data from this participant were excluded from statistical analysis. Post-intervention (T2) data collection at 6, 8, and/or 10 weeks (post-intervention data collection one, two, and three respectively) following the initial data collection. If the participant achieved a NRFS at post-intervention (T2), they were asked to return for a follow-up 4 weeks later (T3).

Twenty-three individuals were screened for this study, but three individuals met exclusion criteria (two with history of forefoot fracture, one with NRFS pattern) (Figure 1). One participant was dropped after developing mononucleosis during the study; these data were excluded. Those who met all of the initial inclusion criteria and none of the exclusion criteria continued with the study (Figure 1). Nineteen participants were included in the final data analysis. Participant demographics and injury details are shown in Table 1. Each participant completed the Numerical Pain Rating Scale [NPRS, scored on a 0–10 scale (Salaffi et al., 2004)], Patient Specific Functional Scale [PSFS, scored on a 0-60 scale (Chatman et al., 1997) with six pre-filled items: walking, running on level ground < 2 miles, running uphill, running downhill, running on level ground > 2 miles, and hopping/jumping], Single Assessment Numerical Evaluation [SANE, scored on a 0–100% scale with 100% equaling full function (Williams et al., 2000)], and the Lower Extremity Functional Scale [LEFS, scored on a 0–80 scale with 80/80 equaling full function (Binkley et al., 1999)] to monitor their self-reported pain level and function during the study.

**Table 1:** Participant demographic, injury/surgery, transition, and re-injury descriptions (*n* = 19).

**Age^†^ (years)**	**Gender**	**History of injury/surgery**	**Time from injury/surgery to study enrollment^†^ (weeks)**	**Days to NRFS transition^†^**	**Sock utilization during runs^†^ (number)**	**NRFS maintained at follow-up?**	**Re-injury within 6 months?**
18	Female	Iliotibial band syndrome	8	48	3	Yes	No
22	Male	Iliotibial band syndrome^#^	24	58	2	Yes	No
40	Female	Iliotibial band syndrome^#^	40	56	12	Yes	No
42	Male	Iliotibial band syndrome^#^	24	62	0	Yes	No
44	Female	Iliotibial band syndrome	12	112	4	Yes	No
18	Male	Mid-shaft tibia stress fracture	12	43	7	Yes	No
20	Female	Mid-shaft tibia stress fracture	28	49	1	Yes	No
19	Female	Open tibia fracture s/p IM nail	32	51	1	Yes	No
21	Male	Patella dislocation^#^	16	42	4	Yes	No
49	Male	Patellofemoral joint OA^#^	22	59	20	No	No
20	Female	Patellofemoral pain syndrome^#^	4	43	9	Yes	No
22	Male	Patellofemoral pain syndrome^#^	13	57	9	Yes	No
42	Male	Plantar fasciitis	32	48	6	Yes	No
20	Male	s/p ACL reconstruction^#^	28	43	7	Yes	No
21	Male	s/p ACL revision^#^	20	57	11	Yes	No
51	Female	s/p Total hip arthroplasty	36	78	17	No	No
18	Female	Medial tibial stress syndrome	6	47	1	Yes	No
24	Male	Medial tibial stress syndrome	20	55	2	Yes	No
37	Female	Medial tibial stress syndrome	4	N/A	6	N/A	No

*NRFS, non-rearfoot strike; IM, intramedullary; OA, osteoarthritis; s/p, status post; ACL, anterior cruciate ligament*.

† *Mean and SD—Age: 28.8 ± 12 years; Time from injury/surgery to study enrollment: 20.1 ± 10.9 weeks; NRFS transition: 56.0 ± 16.6 days; Sock utilization: 6.4 ± 5.7 times*.

# *Unilateral knee injury or surgery in the 12 months prior to study enrollment*.

*N/A, individual did not successfully transition to a NRFS in the allotted time frame*.

### Procedures

#### Initial Data Collection

For the initial data collection (T1), which occurred just prior to the intervention, participants were instructed to perform an initial three-minute warm up run at a self-selected pace (mean 2.91 ± 0.26 m/s) on the single side of a split-belt instrumented treadmill (Bertec, Columbus, Ohio, USA). Kinetic (force) data were collected from three 10-s trials during the fourth minute of running. Participants wore self-selected socks and shoes. Raw kinetic data were collected from the instrumented treadmill at 1,000 Hertz (Hz), filtered at 35 Hz with a fourth order low-pass Butterworth filter, and normalized to body weight using custom code written in MATLAB (The MathWorks, Inc., Natick, MA, USA) to extract AVLR, cadence, peak vGRF, and stance time for both the left and right lower extremity. AVLR was defined as the slope of the vertical ground reaction force curve from 3 to 12% of stance phase (Goss and Gross, 2013), with a foot strike threshold of 50 Newtons. If the impact peak occurred prior to 12% of stance phase, the true impact peak was identified, and this value was used as the second point in the AVLR calculation (Miller et al., 2019). Cadence was calculated by dividing the total time to complete five left and right steps to determine steps *per second* and then multiplying by 60 s to calculate steps *per minute* (Goss and Gross, 2013).

Simultaneously, to detect FSP, two-dimensional sagittal plane video was collected from a Casio High Speed Exilim EX-ZR200 (Tokyo, Japan) digital stationary camera mounted to a Vivitar tripod (80 cm lens height, 80 cm distance, perpendicular to the treadmill; Edison, NJ, USA). Video was sampled at 240 Hz with a resolution of 512 × 384 pixels and shutter speed of 1/1,000s. Windows Movie Maker 7.0 (Microsoft, Redmond, WA, USA) was used to evaluate FSP of the third 10-s running trial to dichotomously categorize FSP into RFS or NRFS running pattern. Location of initial plantar contact observed on video in the posterior one-third of the foot was considered a RFS running pattern; initial plantar contact observed in the anterior two-third of the foot was characterized as a NRFS running pattern. If both anterior and posterior aspects of the foot made initial contact with the ground simultaneously, the foot strike was considered a NRFS pattern. Overall FSP was determined by the largest percentage of foot strikes observed during video analysis. Video analysis of FSP has demonstrated excellent intra- and inter-rater reliability (Esculier et al., 2018; Miller et al., 2018) and good concurrent validity when compared to three-dimensional motion analysis (Esculier et al., 2018).

#### Intervention

Following the initial treadmill data collection (T1), participants received individual instruction on a lower extremity exercise program (HEP; calf stretching, calf foam rolling, plantar fascia stretching, weight shifting, foot tapping, marching on forefeet, toe yoga and knee to chest bridging) in order to increase strength and mobility to improve the runner's capacity for gait retraining (Supplementary Material 3). Participants continued traditional physical therapy for their original lower-extremity injury under the direction and guidance of their original referring physical therapist. In addition to the exercise program, a gradual walk-to-run progression (10 phases, each with specific walk and run intervals that last a duration of 30 min) with strict instructions for appropriate execution was given to all participants to complete on their own. The run progression is a commonly utilized return to run program among military clinicians (Supplementary Material 2) (O'Connor et al., 2001; Miller et al., 2020). Participants were asked to perform the gradual run progression on their own while receiving feedback provided by the instrumented socks. Strict adherence to the walk-to-run progression was encouraged to control feedback dosage and running load. Following NRFS transition and completion of the walk-to-run progression, participants were permitted to return to their normal running routine.

After demonstrating understanding of the HEP and walk-to-run progression, runners were fit with a pair of instrumented socks (Sensoria, Redmond, WA, USA) with three sewn-in textile pressure sensors [heel, head of the first metatarsal, and head of the 5th metatarsal, (Supplementary Material 1)] sampling at 32 Hz. The manufacturer's donning, doffing, and cleaning instructions were given to the participant. The socks determine FSP by detecting heel (RFS) or metatarsal (NRFS) plantar pressure. An anklet containing an accelerometer connects to the sock magnetically and delivers data *via* Bluetooth to a mobile application on the individual's smart device. The smart device provided real-time audio biofeedback on distance covered, pace, FSP, and cadence to the participants while executing the walk-to-run progression (Supplementary Material 2). A faded feedback approach was utilized to encourage internalization of the new movement skill (Willy and Davis, 2013). Walk time and instrumented sock feedback to transition to a NRFS were gradually decreased while runtime was increased. Participants received feedback every minute during phases 1–3 (approximately 6 runs), every 2 min during phases 4–7 (approximately 8 runs), and every 5 min during phases 8–10 (approximately 6 runs) of the walk-to-run progression. This removal of feedback was incorporated to shift dependence from external (instrumented sock) to internal cues to facilitate true motor learning of the non-rearfoot strike pattern (Winstein and Schmidt, 1990). Audio feedback from the smart device provided cues such as, “Try to land more gently” and “You are landing on your heel. Try to lean forward to land on your forefoot.” Cadence was preset to 180 steps/minute (McDougall, 2009; Diebal et al., 2012) and participants were provided cues when they dropped below 180 steps *per minute* for a percentage of the run. The socks have demonstrated moderate FSP reliability and excellent cadence reliability (Stoltenberg et al., 2019). An online account was generated for each participant to track usage of the socks. The participants did not receive any additional instruction on how to transition their foot strike pattern.

#### Post-intervention and Follow-Up Data Collections

Post-intervention data collections (T2) were performed on the instrumented treadmill utilizing the same procedures previously described, to include two-dimensional video analysis for FSP, at the participant's previously self-selected running speed. Participants ran in the same self-selected shoes. Participants returned for a post-intervention data collection at 6, 8, and/or 10 weeks (post-intervention data collection one, two, and three respectively) following the initial data collection. All participants returned for post-intervention one to determine if a NRFS transition had occurred as observed on video analysis. Those who achieved a NRFS transition were asked to return for a follow-up 4 weeks later. Those who did not achieve a NRFS transition were re-instructed in the lower extremity exercise program and walk-to-run program with the use of the instrumented socks, and were asked to return for post-intervention two. If the participant achieved a NRFS at post-intervention two, they were asked to return for a follow-up 4 weeks later (T3). Those who did not achieve a NRFS were re-instructed in the exercise program and walk-to-run program with the use of the instrumented sock, and were asked to return for post-intervention data collection three. If a participant did not transition to a NRFS by the third post-intervention data collection, their contribution to the study was complete (Figure 1).

Participants who successfully transitioned to a NRFS at any of the three post-intervention data collections (T2) were instructed to continue with the previously described exercise program, as well as the walk-to-run progression, but stopped using the socks for biofeedback. A follow-up data collection (T3) was then completed following the same procedures previously described approximately 4 weeks later to assess retention of the NRFS (Figure 1). After the follow-up data collection (T3), their contribution to the study was complete.

A retrospective review of the Department of Defense's electronic medical record was completed 6 months from study enrollment to determine if any medical visits for a new or repeat lower extremity injury occurred.

### Data Reduction

Data from the first five consecutive steps, for the left and right foot, of the third 10-s running trial were used to analyze AVLR, cadence, peak vGRF, and stance time. These data were averaged and carried forward to compare involved vs. uninvolved limbs. For individuals with bilateral complaints (*n* = 4), the more symptomatic side as documented in medical records was used as the involved limb.

### Statistical Analyses

R version 3.4.4 (R Foundation for Statistical Computing, Vienna, Austria) was used to complete statistical analyses. Descriptive statistics were computed to determine the percentage of previous injuries by body region, percentage of individuals who successfully transitioned to a NRFS pattern at each timepoint (T1, T2, and T3) and percentage of individuals who were re-injured over the course of the study. Means, standard deviations and 95% confidence intervals (CIs) were computed for each dependent variable at each time point. A Pearson product-moment correlation was used to assess association between sock usage and days to transition to NRFS pattern among those participants who successfully transitioned to a NRFS. A one-way repeated measures analysis of variance (ANOVA) was computed to compare the mean values of the dependent variables (NPRS, PSFS, SANE, LEFS, AVLR, cadence, peak vGRF, stance time) across each timepoint (T1, T2, T3) for the participants who successfully transitioned to a NRFS. Mauchly's test of sphericity was performed to test for possible violations of sphericity (type I error). When Mauchly's test was significant, a Greenhouse-Geisser correction was applied (Portney and Watkins, 2015). If the RM ANOVA was significant, *post hoc* Bonferroni-Holm pairwise comparisons were computed. A second repeated measures ANOVA was performed on a sub-group of nine participants who had a history of either unilateral knee injury or knee surgery utilizing the same procedures. Values of *p* < 0.05 were considered statistically significant.

## Results

Three individuals did not meet the pre-established inclusion criteria and were excluded from the study (Figure 1). Nineteen individuals (mean age 28.8 ± 12 years; 10 males, 9 females; height 1.7 ± 0.1 m; mass 74.9 ± 11.9 kg; weekly running distance 8.8 ± 7.0 km) met criteria and received intervention (Table 1). The distribution of participant lower extremity injuries by body region was as follows: hip (16%), knee (47%), anterior lower leg (32%), and foot (5%). Nearly all participants (18/19, 95%) transitioned to a non-rearfoot strike (mean 8 ± 2.4) weeks) (Figure 1), and the majority (16/18, 89%) maintained the transition 5-weeks later (mean 5.0 ± 0.8) weeks (Table 2). Most of the participants who transitioned to a NRFS did so at 6-weeks post-intervention (15/18, 83%). The remaining 6 and 11% transitioned at 8- and 10-weeks post-intervention, respectively. Participants who transitioned to a NRFS utilized the socks 6.4 ± 5.7 times over an average of 8 weeks. There was no association observed between sock usage and days to NRFS transition (*p* = 0.72, *r* = 0.14) (Table 2). Additionally, there were no new injuries or repeat injuries observed in the medical record review for the 6 months following study enrollment.

**Table 2:** Rearfoot strike to non-rearfoot strike transition description (*n* = 19).

NRFS transition at post-intervention data collection one: 50.7 ± 7.0 days	15/19 (79%)
NRFS transition at post-intervention data collection two: 69.3 ± 11.8 days	1/4 (25%)
NRFS transition at post-intervention data collection three: 91.3 ± 18.2 days	2/3 (67%)
NRFS transition overall	18/19 (95%)
Maintained NRFS at follow-up data collection: 34.9 ± 5.6 days after transition^#^	16/18 (89%)^*^
Sock usage and days to NRFS transition^†^	*r* = 0.14, *p* = 0.72

*NRFS, non-rearfoot strike*.

*Values are reported as mean and SD from day 0 of the study*.

# *The follow-up data collection is reported as mean number of days and SD from the NRFS transition*.

* *One participant did not transition to a NRFS running pattern and was excluded*.

† *Associated based on Pearson product-moment correlation. Mean sock utilization: 6.4 ± 5.7 times*.

Table 3 demonstrates the results of the RM ANOVA for the variables between three timepoints: (T1, T2, T3). The NPRS, LEFS, and peak vGRF were not significantly different between any of the three time points. Between T1 and T2, pairwise comparisons revealed a significant reduction for AVLR on the involved (*p* = 0.002) and uninvolved limb (*p* = 0.003), significant increase in cadence (*p* = 0.045), and a significant reduction in stance time on the involved (*p* < 0.001) and uninvolved (*p* < 0.001) limb. There were no significant differences between T1 and T2 for the PSFS or SANE. Between T2 and T3, a significant increase on the PSFS (*p* = 0.04) and the SANE (*p* = 0.04) were observed, indicating improved function. No significant difference was noted between T2 and T3 for AVLR, cadence, or stance time. Between T1 and T3, significant differences were noted for an increase in PSFS (*p* = 0.04), a reduction in AVLR on the involved (*p* = 0.008) and uninvolved (*p* = 0.01) limb, and a reduction in stance time on the involved (*p* < 0.001) and uninvolved (*p* < 0.001) limb. There were no significant differences noted between T1 and T3 for the SANE or cadence.

**Table 3:** Repeated measures analysis of variance pairwise comparison with Holm correction for participants who transitioned to a NRFS running pattern (*n* = 18^#^).

**Variable**	**Limb**	**Initial data collection (T1)**	**Post-intervention data collection^†^ (T2)**	**Follow-up data collection^**^ϕ^**^ (T3)**	**Notes**
NPRS		0.00 (0.00–0.00)	1.25 (0.23–2.27)	0.58 (0.01–1.15)	T1–T2: *p* = 0.06 T2–T3: *p* = 0.15 T1–T3: *p* = 0.09
PSFS		49.67 (43.38–55.86)	51.78 (47.35–56.21)	55.83 (52.36–59.30)	T1–T2: *p* = 0.41 T2–T3: *p* = 0.04^*^ T1–T3: *p* = 0.04^*^
SANE		85.00 (77.86–92.14)	87.44 (80.32–94.56)	91.58 (85.02–98.14)	T1–T2: *p* = 0.57 T2–T3: *p* = 0.04^*^ T1–T3: *p* = 0.21
LEFS		74.67 (72.04–77.30)	75.72 (72.97–78.48)	76.22 (73.06–79.38)	T1–T2: *p* = 0.49 T2–T3: *p* = 0.49 T1–T3: *p* = 0.49
Average vertical loading rate (BW/s)	Involved	54.72 (48.17–61.26)	38.73 (33.72–43.75)	38.41 (30.31–46.51)	T1–T2: *p* = 0.002^*^ T2–T3: *p* = 0.91 T1–T3: *p* = 0.008^*^
	Uninvolved	55.12 (48.81–61.43)	38.90 (33.93–43.87)	41.62 (35.76–47.47)	T1–T2: *p* = 0.003^*^ T2–T3: *p* = 0.10 T1–T3: *p* = 0.01^*^
Cadence (steps/min)	Mean of involved and uninvolved	168.51 (164.95–172.08)	173.30 (170.30–176.30)	171.57 (168.28–174.86)	T1–T2: *p* = 0.045^*^ T2–T3: *p* = 0.19 T1–T3: *p* = 0.08
Peak vertical GRF (BW)	Involved	2.26 (2.14–2.38)	2.35 (2.23–2.46)	2.38 (2.26–2.50)	T1–T2: *p* = 0.06 T2–T3: *p* = 0.14 T1–T3: *p* = 0.06
	Uninvolved	2.31 (2.19–2.43)	2.36 (2.25–2.48)	2.40 (2.27–2.54)	T1–T2: *p* = 0.25 T2–T3: *p* = 0.25 T1–T3: *p* = 0.17
Stance time (s)	Involved	0.27 (0.26–0.28)	0.24 (0.23–0.25)	0.25 (0.23–0.26)	T1–T2: *p* < 0.001^*^ T2–T3: *p* = 0.30 T1–T3: *p* < 0.001^*^
	Uninvolved	0.27 (0.26–0.28)	0.25 (0.24–0.26)	0.25 (0.24–0.26)	T1–T2: *p* < 0.001^*^ T2–T3: *p* = 0.42 T1–T3: *p* < 0.001^*^

*NRFS, non-rearfoot strike; NPRS, numerical pain rating scale; PSFS, patient specific functional scale; SANE, single assessment numerical evaluation; LEFS, lower extremity functional scale; GRF, ground reaction force; BW, body weights*.

*These data are reported as mean and 95% confidence interval of the involved and uninvolved lower extremity, unless otherwise noted*.

# *One participant did not transition to a NRFS running pattern and was excluded from these data*.

† *Mean days from initial to post-intervention data collection: 56.0 ± 16.6 days*.

ϕ *Mean days from post-intervention to follow-up data collection: 34.9 ± 5.6 days*.

* *Denotes significance (p <0.05)*.

A sub-analysis was performed on nine individuals who had a history of either unilateral knee injury or knee surgery (involved limb) in the 12 months prior to study enrollment. Supplementary Material 4 shows the results of the RM ANOVA at the same three time points as previously discussed. All nine individuals in the sub-analysis initially transitioned to a NRFS (100%) and 8/9 or 89% maintained the NRFS transition at follow-up (T3). The NPRS, functional outcome measures, AVLR on the uninvolved limb, and peak vGRF did not demonstrate a significant difference between time points. Between T1 and T2, pairwise comparisons revealed a reduction in AVLR on the involved limb (*p* = 0.04), as well as reductions in stance time on both the involved (*p* < 0.001) and uninvolved limb (*p* = 0.002). No significant differences were noted between T1 and T2 for cadence. There were no significant differences between T2 and T3 for AVLR, cadence, or stance time. Between T1 and T3, a reduction in AVLR was observed for the involved limb (*p* = 0.04), an increase in cadence (*p* = 0.01), and a reduction in stance time on both the involved (*p* < 0.001) and uninvolved (*p* = 0.003) limb.

## Discussion

The purpose of this case series was to examine the effectiveness of an instrumented sock to transition previously injured runners to a NRFS running pattern and subsequently examine the interventions effect on pain scores, patient-reported outcome measures, and running biomechanics. In accordance with our original hypotheses, greater than 90% of the participants successfully transitioned from a RFS to a NRFS with the use of the instrumented socks (Figure 1) and we observed accompanied improvements in patient-self reported function, reduced AVLR bilaterally, and reduced stance time bilaterally. Our findings demonstrate that gait retraining FSP using instrumented socks can improve several key biomechanical factors related to injury risk.

In the present study, a higher proportion of individuals transitioned to a NRFS pattern (90%) compared to two previous reports; an in-field NRFS transition study using wearable technology in healthy individuals (75%) (Chan et al., 2020a) and a laboratory-based NRFS transition study using real-time visual feedback of FSP in healthy individuals (40%) (Chan et al., 2020b). Two previous studies achieved an even higher success rate (100%) utilizing in-clinic verbal feedback to transition previously injured runners to a NRFS pattern (Roper et al., 2016; Miller et al., 2020). Previously injured runners may be more motivated to alter gait mechanics than healthy runners to decrease pain or improve function. Additionally, the ideal feedback method (verbal, visual, and audio) and technology needed to successfully alter an individual's FSP is unknown and further research is required to address the efficacy and effectiveness of different feedback mechanisms for different patient populations. The present study adds to the current literature, indicating that real-time audio biofeedback provided by an instrumented sock was effective in modifying the FSP of previously injured military service members.

The transition of FSP to a NRFS resulted in improvements in running kinetics. Confirming our hypothesis, we observed a reduction in AVLR on the involved (30%) and uninvolved (24%) limbs from initial (T1) to follow-up (T3). The large magnitude and clinically meaningful reductions in AVLR observed in the current study correspond to the 40–50% reductions previously reported in the literature following NRFS transition (Cheung and Davis, 2011; Crowell and Davis, 2011; Futrell et al., 2020). The current study utilized both an internal attentional focus strategy (“Try to land more gently”) and external strategy (“You are landing on your heel. Try to lean forward to land on your forefoot,” cadence feedback) to achieve changes in FSP. The discrepancy in percent AVLR reduction among studies following a NRFS transition may be explained by the different feedback mechanisms provided to participants (Wulf and Prinz, 2001; Moore et al., 2019).

A multitude of interventions have been proposed for the treatment of running injuries to include therapeutic exercise, foot orthoses, alterations in footwear, and gait retraining. In one report, runners who transitioned from a RFS to a NRFS pattern demonstrated a two-fold greater reduction in impact forces than the use of cushioned soles, foot orthoses, and shock attenuating insoles, leading to the suggestion that a runner's ability to manipulate their mechanics may lead to greater changes than external devices (footwear, orthoses) (Crowell and Davis, 2011). The data from the current study support a similar magnitude reduction in impact forces (AVLR) following a NRFS gait retraining intervention using instrumented socks. Technology such as this may increase access to medical care and reduce medical costs following injury by decreasing the number of required in-clinic visits.

It is well established that running with a NRFS compared to a RFS pattern redistributes the mechanical load associated with running from the knee to the ankle and calf musculature (Kulmala et al., 2013). For this reason, gait retraining FSP and to reduce impact loading have been suggested as interventions for patients with PFPS (Roper et al., 2016; Davis et al., 2020). In the current study, a sub-analysis revealed a reduction in AVLR from initial (T1) to follow-up (T3) in the involved (29%) and uninvolved (27%) limbs of participants recovering from unilateral knee injury or surgery. This subset of individuals also demonstrated the greatest increase in cadence and greatest reduction in AVLR on both limbs (31%) at the post-intervention data collection (T2). Our observation is in agreement with Huang et al., who demonstrated that healthy individuals transitioning to a NRFS combined with increased cadence achieved greater reductions in AVLR than FSP manipulation alone (Huang et al., 2019). Overall, we achieved favorable reductions in AVLR in those with knee complaints with a NRFS transition using an instrumented sock. Our results contribute to an evolving body of literature advocating for an injury-specific approach to biomechanical risk factors for injury (Johnson et al., 2020).

At follow-up, the participants demonstrated reduced ground stance time during running. Greater contact times have been previously associated with increased running-related injury risk in Soldiers (Weart et al., 2020) and increased plantar loads and impulse during running (Wellenkotter et al., 2014). A NRFS transition using instrumented socks successfully reduced this potential risk factor for injury and the reduction was maintained at follow-up.

In further support of our hypotheses, cadence increased 2.8% from initial (T1) to post-intervention (T2), but this increase was not maintained at follow-up (T3) (Table 3, Supplementary Material 4). A cadence increase of 5–10% has been shown to reduce energy absorption of the lower-extremities (Heiderscheit et al., 2011) and reduce ground reaction forces during running (Schubert et al., 2014; Huang et al., 2019; Futrell et al., 2020). Furthermore, it has been reported to be more natural and easier to transition FSP using a combined gait modification involving a NRFS and increased cadence than to transition using an isolated gait modification of NRFS alone (Huang et al., 2019). Although the primary intervention for the current study was not cadence manipulation, during running the participants devices provided auditory feedback whenever cadence dropped below 180 steps *per minute*. Despite returning to their initial cadence, a majority of the participants in the current study maintained the NRFS pattern and reductions in AVLR at follow-up. This observation supports the notion that while cadence may help facilitate a more natural modification of FSP, it is not necessary for longer-term NRFS adoption and AVLR reduction.

Reductions in pain and improvements in function following gait retraining have been well documented. In the current study, PSFS scores improved by a mean of 1.1 points from initial (T1) to follow-up (T3), but this change did not exceed the minimal clinically important difference for a small change of 1.3 points (Abbott and Schmitt, 2014). The improvement in function noted is consistent with two previous reports following gait retraining in individuals recovering from PFPS (Noehren et al., 2011) and from lower extremity running related injuries (Miller et al., 2020). Participants in the current study also remained injury free (repeat or new) 6 months after enrollment. Despite the success of our participants, clinicians implementing gait retraining should be aware of the possible risks of altering FSP. A NRFS exposes runners to greater injury risk in the ankle and foot (Kulmala et al., 2013; Chen et al., 2019). To mitigate injury risk in the current study we excluded individuals with previous foot fracture, included aspects of a commonly used gait retraining strengthening program for the feet and lower legs (Futrell et al., 2020) and implemented a gradual walk-to-run progression (Miller et al., 2020).

In the current study a faded feedback program using auditory cues was employed. Auditory cues are more effective than visual feedback (Sigrist et al., 2013) and faded feedback programs have been successfully implemented in several gait retraining studies to optimize motor learning (Roper et al., 2016). Interestingly, the two participants who did not maintain a NRFS at follow-up (T3) were also the oldest participants (male, age 49; female, age 51) and took the longest to initially transition albeit using the socks more than any other individuals in the study. Future research should seek to understand the ideal feedback approaches and parameters needed to achieve optimal motor learning for varying ages, skills, and fitness levels.

### Limitations

Several limitations to the current study must be considered. While the smart device application saved data from each event, the application could not differentiate between walking and running, preventing any analysis to identify timing of the NRFS transition. Participants were instructed in the details of an exercise program, but compliance is unknown. Additionally, the small sample size and lack of control group limits conclusion of treatment effects and did not account for improvements in biomechanics and function that may have occurred naturally over time. Heterogeneity of injury type including duration and location of injury limits global generalizability; however, it is noteworthy that the majority of participants with various injury types transitioned FSP without any in-clinic feedback, demonstrated reductions in AVLR, and none reported new or repeat injuries within 6 months of study enrollment. Finally, it is important to note that the instrumented socks were not the only aspect of the intervention and our study design did not account for the potential effects the walk-to-run progression and exercise program could have had on the participants NRFS transition. However, there is substantial evidence that strengthening, and movement training do not independently influence running biomechanics (Willy and Davis, 2011; Brindle et al., 2020; Foch et al., 2020). Therefore, the authors are confident that the instrumented sock was responsible for the NRFS transition and this primary outcome was not influenced by the exercise program or walk-to-run progression. Larger-scale, randomized control trials with diagnosis-specified running-related injuries are needed to further confirm these findings.

## Conclusion

With technology provided foot strike pattern and cadence auditory feedback from instrumented socks, approximately 90% of participants successfully transitioned to a NRFS pattern and maintained these running adaptations 5-weeks later. In addition, several biomechanical variables associated with an increased injury risk during running (AVLR, stance time) were reduced following transition to a NRFS running pattern and no participants were re-injured over the duration of the study. Our results suggest that wearable technology with foot strike and cadence auditory faded feedback may assist previously injured military service members to transition from a RFS to a NRFS running pattern and subsequently improve their running biomechanics and future injury risk.

## Data Availability Statement

The raw data supporting the conclusions of this article will be made available by the authors, without undue reservation.

## Ethics Statement

This study involving human participants was reviewed and approved by the Keller Army Community Hospital Institutional Review Board. The patients/participants provided their written informed consent to participate in this study. Written informed consent was obtained from the individuals for the publication of any potentially identifiable images or data included in this article.

## Author Contributions

DG, DW, EM, and ES designed the research protocol. DW, EM, AW, ES, and GF performed data collection. GF wrote code for data analysis. DG, DW, EM, and GF analyzed and interpreted the data. All authors contributed to the final version of the manuscript.

## Conflict of Interest

The authors declare that the research was conducted in the absence of any commercial or financial relationships that could be construed as a potential conflict of interest.

## References

[B1] Abbott J. H. Schmitt J. (2014). Minimum important differences for the patient-specific functional scale, 4 region-specific outcome measures, and the numeric pain rating scale. J. Orthop. Sports Phys. Ther. 44, 560–564. 10.2519/jospt.2014.5248 24828475

[B2] Almeida M. O. Davis I. S. Lopes A. D. (2015). Biomechanical differences of foot-strike patterns during running: a systematic review with meta-analysis. J. Orthop. Sports Phys. Ther. 45, 738–755. 10.2519/jospt.2015.6019 26304644

[B3] Barton C. Bonanno D. Carr J. Neal B. Malliaras P. Franklyn-Miller A.. (2016). Running retraining to treat lower limb injuries: a mixed-methods study of current evidence synthesised with expert opinion. Br. J. Sports Med. 50, 513–526. 10.1136/bjsports-2015-095278 26884223

[B4] Binkley J. M. Stratford P. W. Lott S. A. Riddle D. L. Network N. A. O. R. R. (1999). The ower ExtreLmity Functional Scale (LEFS): scale development, measurement properties, and clinical application. Phys. Ther. 79, 371–383. 10201543

[B5] Brindle R. A. Ebaugh D. D. Willson J. D. Finley M. A. Shewokis P. A. Milner C. E. (2020). Relationships of hip abductor strength, neuromuscular control, and hip width to femoral length ratio with peak hip adduction angle in healthy female runners. J. Sports Sci. 38, 2291–2297. 10.1080/02640414.2020.1779489 32543341

[B6] Ceyssens L. Vanelderen R. Barton C. Malliaras P. Dingenen B. (2019). Biomechanical risk factors associated with running-related injuries: a systematic review. Sports Med. 49, 1095–1115. 10.1007/s40279-019-01110-z 31028658

[B7] Chan P. P. Chan Z. Y. Au I. P. Lam B. M. Lam W. Cheung R. T. (2020a). Biomechanical effects following footstrike pattern modification using wearable sensors. J. Sci. Med. Sport. 24,30–35. 10.1016/j.jsams.2020.05.019 32553447

[B8] Chan Z. Y. Zhang J. H. Ferber R. Shum G. Cheung R. T. (2020b). The effects of midfoot strike gait retraining on impact loading and joint stiffness. Phys. Ther. Sport 42, 139–145. 10.1016/j.ptsp.2020.01.011 31995786

[B9] Chatman A. B. Hyams S. P. Neel J. M. Binkley J. M. Stratford P. W. Schomberg A.. (1997). The patient-specific functional scale: measurement properties in patients with knee dysfunction. Phys. Ther. 77, 820–829. 10.1093/ptj/77.8.820 9256870

[B10] Chen T. L.-W. Wong D. W.-C. Wang Y. Lin J. Zhang M. (2019). Foot arch deformation and plantar fascia loading during running with rearfoot strike and forefoot strike: a dynamic finite element analysis. J. Biomech. 83, 260–272. 10.1016/j.jbiomech.2018.12.007 30554818

[B11] Cheung R. T. Davis I. S. (2011). Landing pattern modification to improve patellofemoral pain in runners: a case series. J. Orthop. Sports Phys. Ther. 41, 914–919. 10.2519/jospt.2011.3771 22031595

[B12] Crowell H. P. Davis I. S. (2011). Gait retraining to reduce lower extremity loading in runners. Clin. Biomech. (Bristol, Avon) 26, 78–83. 10.1016/j.clinbiomech.2010.09.003 20888675 PMC3014399

[B13] Davis I. S. Tenforde A. S. Neal B. S. Roper J. L. Willy R. W. (2020). Gait retraining as an intervention for patellofemoral pain. Curr. Rev. Musculoskelet. Med. 13, 103–114. 10.1007/s12178-020-09605-3 32170556 PMC7083994

[B14] DeJong A. F. Hertel J. (2018). Gait-training devices in the treatment of lower extremity injuries in sports medicine: current status and future prospects. Expert. Rev. Med. Devices 15, 891–909. 10.1080/17434440.2018.1551130 30466335

[B15] Diebal A. R. Gregory R. Alitz C. Gerber J. P. (2012). Forefoot running improves pain and disability associated with chronic exertional compartment syndrome. Am. J. Sports Med. 40, 1060–1067. 10.1177/0363546512439182 22427621

[B16] Esculier J. F. Silvini T. Bouyer L. J. Roy J. S. (2018). Video-based assessment of foot strike pattern and step rate is valid and reliable in runners with patellofemoral pain. Phys. Ther. Sport 29, 108–112. 10.1016/j.ptsp.2016.11.003 28666810

[B17] Foch E. Brindle R. A. Milner C. E. (2020). Weak associations between hip adduction angle and hip abductor muscle activity during running. J. Biomech. 110:109965. 10.1016/j.jbiomech.2020.109965 32827779

[B18] Futrell E. E. Gross K. D. Reisman D. Mullineaux D. R. Davis I. S. (2020). Transition to forefoot strike reduces load rates more effectively than altered cadence. J. Sport Health Sci. 9, 248–257. 10.1016/j.jshs.2019.07.006 32444149 PMC7242218

[B19] Goss D. L. Gross M. T. (2013). A comparison of negative joint work and vertical ground reaction force loading rates in Chi runners and rearfoot-striking runners. J. Orthop. Sports Phys. Ther. 43, 685–692. 10.2519/jospt.2013.4542 24256170

[B20] Goss D. L. Lewek M. Yu B. Ware W. B. Teyhen D. S. Gross M. T. (2015). Lower extremity biomechanics and self-reported foot-strike patterns among runners in traditional and minimalist shoes. J. Athl. Train. 50, 603–611. 10.4085/1062-6050.49.6.06 26098391 PMC4527444

[B21] Hasegawa H. Yamauchi T. Kraemer W. J. (2007). Foot strike patterns of runners at the 15-km point during an elite-level half marathon. J. Strength Cond. Res. 21, 888–893. 10.1519/00124278-200708000-00040 17685722

[B22] Hauret K. G. Bedno S. Loringer K. Kao T. C. Mallon T. Jones B. H. (2015). Epidemiology of exercise- and sports-related injuries in a population of young, physically active adults: a survey of military servicemembers. Am. J. Sports Med. 43, 2645–2653. 10.1177/0363546515601990 26378031

[B23] Heiderscheit B. C. Chumanov E. S. Michalski M. P. Wille C. M. Ryan M. B. (2011). Effects of step rate manipulation on joint mechanics during running. Med. Sci. Sports Exerc. 43, 296–302. 10.1249/MSS.0b013e3181ebedf4 20581720 PMC3022995

[B24] Huang Y. Xia H. Chen G. Cheng S. Cheung R. T. Shull P. B. (2019). Foot strike pattern, step rate, and trunk posture combined gait modifications to reduce impact loading during running. J. Biomech. 86, 102–109. 10.1016/j.jbiomech.2019.01.058 30792072

[B25] Johnson C. D. Tenforde A. S. Outerleys J. Reilly J. Davis I. S. (2020). Impact-related ground reaction forces are more strongly associated with some running injuries than others. Am. J. Sports Med. 48, 3072–3080. 10.1177/0363546520950731 32915664

[B26] Kulmala J. P. Avela J. Pasanen K. Parkkari J. (2013). Forefoot strikers exhibit lower running-induced knee loading than rearfoot strikers. Med. Sci. Sports Exerc. 45, 2306–2313. 10.1249/MSS.0b013e31829efcf7 23748735

[B27] Lieberman D. E. Venkadesan M. Werbel W. A. Daoud A. I. D'Andrea S. Davis I. S.. (2010). Foot strike patterns and collision forces in habitually barefoot versus shod runners. Nature 463, 531–535. 10.1038/nature08723 20111000

[B28] McDougall C. (2009). Born to Run. New York, NY: Alfred A. Knopf.

[B29] Miller E. Morris J. Watson D. Goss D. (2018). A reliability comparison of different methods for detecting step rate and foot strike pattern in runners using two-dimensional video. Universal J. Public Health 6, 366–371. 10.13189/ujph.2018.060608

[B30] Miller E. Weart A. Freisinger G. M. Brindle R. A. Goss D. (2019). A novel method for evaluating loading rate during running regardless of impact peak, in Combined International Society of Biomechanics and American Society of Biomechanics Meeting (Calgary, AB).

[B31] Miller E. M. Crowell M. S. Morris J. B. Mason J. S. Zifchock R. Goss D. L. (2020). Gait Retraining Improves Running Impact Loading and Function in Previously Injured US Military Cadets: A Pilot Study. Mil. Med. 10.1093/milmed/usaa383 [Epub ahead of print]. 33215669

[B32] Molloy J. M. (2016). Factors influencing running-related musculoskeletal injury risk among U.S. military recruits. Mil. Med. 181, 512–523. 10.7205/MILMED-D-15-00143 27244060

[B33] Moore I. S. Phillips D. J. Ashford K. J. Mullen R. Goom T. Gittoes M. R. (2019). An interdisciplinary examination of attentional focus strategies used during running gait retraining. Scand. J. Med. Sci. Sports 29, 1572–1582. 10.1111/sms.13490 31149751

[B34] Morris J. B. Goss D. L. Miller E. M. Davis I. S. (2020). Using real-time biofeedback to alter running biomechanics: a randomized controlled trial. Transl. Sports Med. 3, 63–71. 10.1002/tsm2.110

[B35] Noehren B. Scholz J. Davis I. (2011). The effect of real-time gait retraining on hip kinematics, pain and function in subjects with patellofemoral pain syndrome. Br. J. Sports Med. 45, 691–696. 10.1136/bjsm.2009.069112 20584755

[B36] O'Connor F. G. Wilder R. P. Nirschl R. (2001). Textbook of Running Medicine. New York: NY: McGraw-Hill.

[B37] Phanpho C. Rao S. Moffat M. (2019). Immediate effect of visual, auditory and combined feedback on foot strike pattern. Gait. Posture 74, 212–217. 10.1016/j.gaitpost.2019.09.016 31561119

[B38] Pohl M. B. Hamill J. Davis I. S. (2009). Biomechanical and anatomic factors associated with a history of plantar fasciitis in female runners. Clin. J. Sport Med. 19, 372–376. 10.1097/JSM.0b013e3181b8c270 19741308

[B39] Portney L. G. Watkins M. P. (2015). Foundations of Clinical Research: Applications to Practice. Philadelphia, PA: FA Davis Company.

[B40] Roper J. L. Harding E. M. Doerfler D. Dexter J. G. Kravitz L. Dufek J. S.. (2016). The effects of gait retraining in runners with patellofemoral pain: a randomized trial. Clin. Biomech. 35, 14–22. 10.1016/j.clinbiomech.2016.03.010 27111879

[B41] Salaffi F. Stancati A. Silvestri C. A. Ciapetti A. Grassi W. (2004). Minimal clinically important changes in chronic musculoskeletal pain intensity measured on a numerical rating scale. Eur. J. Pain 8, 283–291. 10.1016/j.ejpain.2003.09.004 15207508

[B42] Schubert A. G. Kempf J. Heiderscheit B. C. (2014). Influence of stride frequency and length on running mechanics: a systematic review. Sports Health 6, 210–217. 10.1177/1941738113508544 24790690 PMC4000471

[B43] Sigrist R. Rauter G. Riener R. Wolf P. (2013). Augmented visual, auditory, haptic, and multimodal feedback in motor learning: a review. Psychon. Bull. Rev. 20, 21–53. 10.3758/s13423-012-0333-8 23132605

[B44] Stoltenberg B. E. Miller E. M. Dolbeer J. A. Pickens B. B. Goss D. L. (2019). Validity of an instrumented sock and on-shoe sensor to provide biometric feedback to runners. Footwear Sci. 11, 147–152. 10.1080/19424280.2019.1614098

[B45] Tate J. J. Milner C. E. (2010). Real-time kinematic, temporospatial, and kinetic biofeedback during gait retraining in patients: a systematic review. Phys. Ther. 90, 1123–1134. 10.2522/ptj.20080281 20558567

[B46] van der Worp H. Vrielink J. W. Bredeweg S. W. (2016). Do runners who suffer injuries have higher vertical ground reaction forces than those who remain injury-free? A systematic review and meta-analysis. Br. J. Sports Med. 50, 450–457. 10.1136/bjsports-2015-094924 26729857

[B47] van Gent B. R. Siem D. D. van Middelkoop M. van Os T. A. Bierma-Zeinstra S. S. Koes B. B. (2007). Incidence and determinants of lower extremity running injuries in long distance runners: a systematic review. Br. J. Sports Med. 41, 469–480. 10.1136/bjsm.2006.033548 17473005 PMC2465455

[B48] Van Hooren B. Goudsmit J. Restrepo J. Vos S. (2020). Real-time feedback by wearables in running: current approaches, challenges and suggestions for improvements. J. Sports Sci. 38, 214–230. 10.1080/02640414.2019.1690960 31795815

[B49] Vannatta C. N. Kernozek T. W. (2015). Patellofemoral joint stress during running with alterations in foot strike pattern. Med. Sci. Sports Exerc. 47, 1001–1008. 10.1249/MSS.0000000000000503 25202853

[B50] Warne J. P. Smyth B. P. Fagan J. O. C. Hone M. E. Richter C. Nevill A. M.. (2017). Kinetic changes during a six-week minimal footwear and gait-retraining intervention in runners. J. Sports Sci. 35, 1538–1546. 10.1080/02640414.2016.1224916 27571390

[B51] Weart A. N. Miller E. M. Ford K. R. Brindle R. A. Goss D. L. (2020). Can wearable technology prospectively identify contributors to running related injuries in active duty soldiers?, in Military Health System Research Symposium. Available at: https://mhsrs.amedd.army.mil/submissions/SitePages/Accepted-Abstracts.aspx (accessed August 27, 2020).

[B52] Wellenkotter J. Kernozek T. W. Meardon S. Suchomel T. (2014). The effects of running cadence manipulation on plantar loading in healthy runners. Int. J. Sports Med. 35, 779–784. 10.1055/s-0033-1363236 24595812

[B53] Williams G. N. Taylor D. C. Gangel T. J. Uhorchak J. M. Arciero R. A. (2000). Comparison of the single assessment numeric evaluation method and the lysholm score. Clin. Orthop. Relat. Res. 373, 184–192. 10.1097/00003086-200004000-00022 10810476

[B54] Willy R. W. Buchenic L. Rogacki K. Ackerman J. Schmidt A. Willson J. D. (2016). In-field gait retraining and mobile monitoring to address running biomechanics associated with tibial stress fracture. Scand. J. Med. Sci. Sports 26, 197–205. 10.1111/sms.12413 25652871

[B55] Willy R. W. Davis I. S. (2011). The effect of a hip-strengthening program on mechanics during running and during a single-leg squat. J. Orthop. Sports Phys. Ther. 41, 625–632. 10.2519/jospt.2011.3470 21765220

[B56] Willy R. W. Davis I. S. (2013). Varied response to mirror gait retraining of gluteus medius control, hip kinematics, pain, and function in 2 female runners with patellofemoral pain. J. Orthop. Sports Phys. Ther. 43, 864–874. 10.2519/jospt.2013.4516 24175611

[B57] Winstein C. J. Schmidt R. A. (1990). Reduced frequency of knowledge of results enhances motor skill learning. J. Exp. Psychol. Learn. Mem. Cogn. 16:677. 10.1037/0278-7393.16.4.677

[B58] Wulf G. Prinz W. (2001). Directing attention to movement effects enhances learning: a review. Psychon. Bull. Rev. 8, 648–660. 10.3758/BF03196201 11848583

[B59] Xu Y. Yuan P. Wang R. Wang D. Liu J. Zhou H. (2020). Effects of foot strike techniques on running biomechanics: a systematic review and meta-analysis. Sports Health 13, 71–77. 10.1177/1941738120934715 32813597 PMC7734358

[B60] Zadpoor A. A. Nikooyan A. A. (2011). The relationship between lower-extremity stress fractures and the ground reaction force: a systematic review. Clin. Biomech. (Bristol, Avon) 26, 23–28. 10.1016/j.clinbiomech.2010.08.005 20846765

[B61] Zifchock R. A. Davis I. Hamill J. (2006). Kinetic asymmetry in female runners with and without retrospective tibial stress fractures. J. Biomech. 39, 2792–2797. 10.1016/j.jbiomech.2005.10.003 16289516

