# Supplementary material for: Wearable Technology May Assist in Retraining Foot Strike Patterns in Previously Injured Military Service Members: A Prospective Case Series
Source: Front Sports Act Living. 2021 Feb 26;3:630937. doi: 10.3389/fspor.2021.630937 (PMC7952986; doi:10.3389/fspor.2021.630937)
Supplement: Supplementary file 1 [file Data_Sheet_1.PDF]

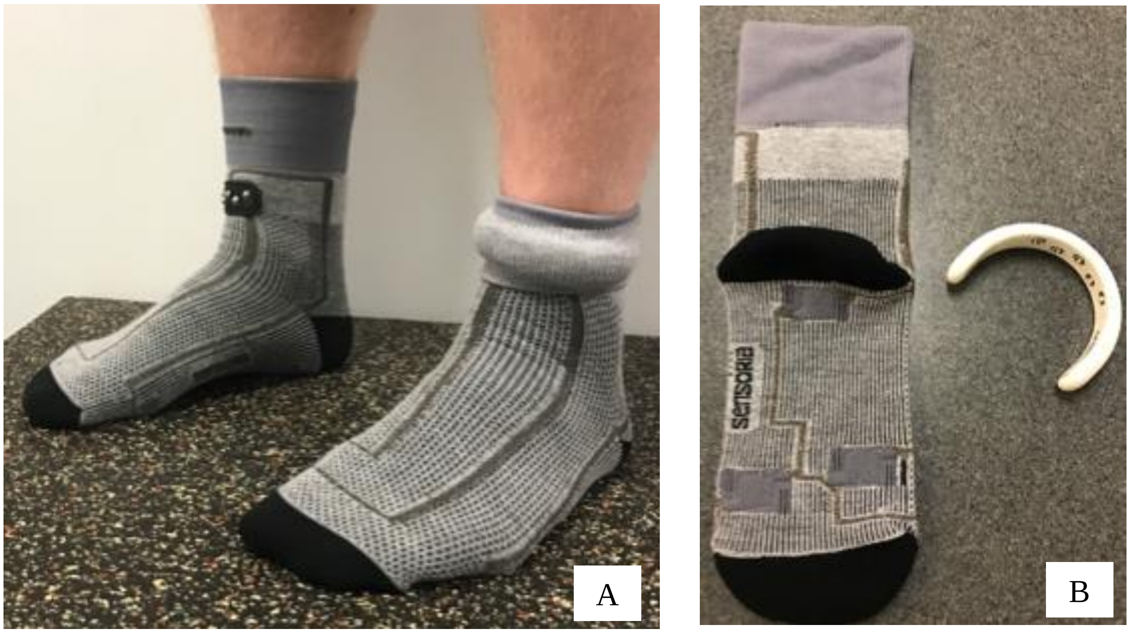

Supplement 1. Instrumented socks.

(**A**) Instrumented socks with the accelerometer on the left sock. (**B**) Plantar aspect of the instrumented socks highlighting the three electronic sensors at the head of the 1st and 5th metatarsals and the calcaneus. Accelerometer is at right.
