# Supplementary material for: Wearable Technology May Assist in Retraining Foot Strike Patterns in Previously Injured Military Service Members: A Prospective Case Series
Source: Front Sports Act Living. 2021 Feb 26;3:630937. doi: 10.3389/fspor.2021.630937 (PMC7952986; doi:10.3389/fspor.2021.630937)

Supplement 2: Walk-to-Run Progression

# Walk-to-Run Progression

<u>Instructions:</u>

1. Begin this program only if you are able to walk two miles in 35 minutes without pain.
2. All participants should begin at phase 1 and progress as instructed (even if you are currently running).
3. Perform the walk/run <u>every other day only</u>. Do not run two days in a row.
4. Perform at a self-selected pace on a <u>level surface</u> - no hills. Walk/running on a treadmill will allow easy monitoring of not only time/distance, but also the feedback from the Sensoria® app on your cell phone or tablet device.
5. Please utilize good running shoes that are not more than 6-9 months old, if you were running or walking in them.
6. Discuss with your medical provider if you are unsure if your shoes contributed to your previous injury. If your shoes did contribute, consider changing your running shoes.
7. If you experience increased pain, swelling, or stiffness during or after a phase, please stop. Symptoms may not arise until the following day. Please do not run again until your symptoms have resolved and when you do return to running, return to the last phase which was pain free. If you are in doubt or your pain, swelling, stiffness does not resolve, please follow up with your medical provider.
8. Perform each phase at least twice. You may progress to the next phase if you do not experience any pain, swelling, or stiffness during or after the walk/run performance.
9. After completion of phase 10, if greater running distance is desired, progress as tolerated.
10. Please stretch/use ice as needed.
11. Set your feedback from the Sensoria® app on your cell phone or tablet device as instructed.

Begin each session with a 10 minute warm-up of walking, biking, or using the elliptical.

| Phase | Sensoria® Status Updates | Walk (min) | Run (min) | Repeat | Total Time (min) | Performed |
|---|---|---|---|---|---|---|
| 1 | Every 1 Minute | 5 | 1 | x5 | 30 | □ □ |
| 2 | Every 1 Minute | 4 | 2 | x5 | 30 | □ □ |
| 3 | Every 1 Minute | 3 | 3 | x5 | 30 | □ □ |
| 4 | Every 2 Minutes | 2 | 4 | x5 | 30 | □ □ |
| 5 | Every 2 Minutes | 1 | 5 | x5 | 30 | □ □ |
| 6 | Every 2 Minutes | 5 | 10 | x2 | 30 | □ □ |
| 7 | Every 2 Minutes | 0 | 15 | x1 | 15 | □ □ |
| 8 | Every 5 Minutes | 0 | 20 | x1 | 20 | □ □ |
| 9 | Every 5 Minutes | 0 | 25 | x1 | 25 | □ □ |
| 10 | Every 5 Minutes | 0 | 30 | x1 | 30 | □ □ |

To update the feedback from the Sensoria® app:

1) Click on notifications

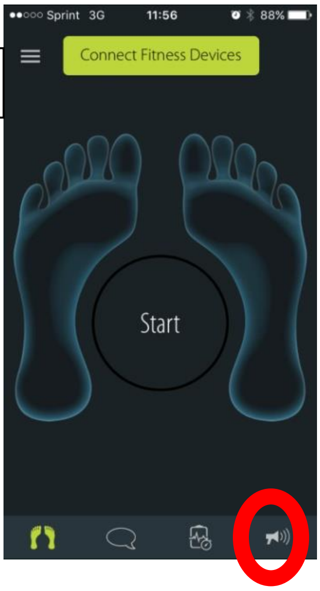


2) Click on status updates

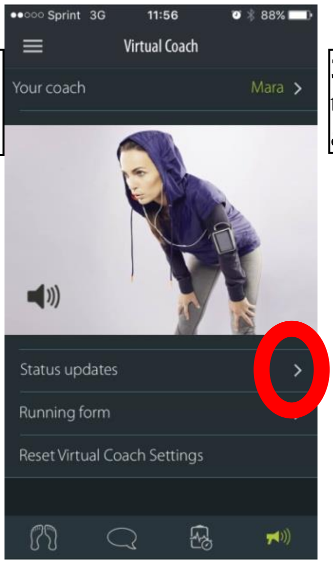


3) Adjust time as above

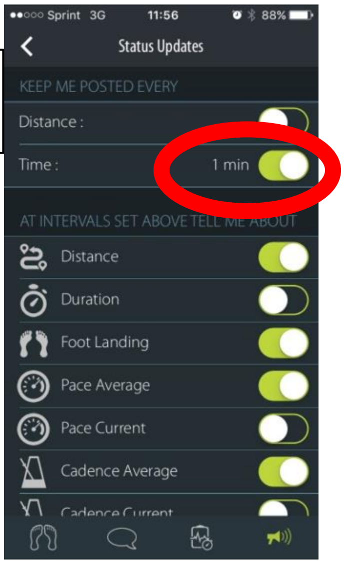

Supplement: Supplementary file 2 [file Data_Sheet_2.PDF]
