# Supplementary material for: Wearable Technology May Assist in Retraining Foot Strike Patterns in Previously Injured Military Service Members: A Prospective Case Series
Source: Front Sports Act Living. 2021 Feb 26;3:630937. doi: 10.3389/fspor.2021.630937 (PMC7952986; doi:10.3389/fspor.2021.630937)
Supplement: Supplementary file 3 [file Data_Sheet_3.PDF]

Supplement 3: Lower extremity exercise program

Exercises to Assist in Changing Your Running Form

This **LOWER EXTREMITY EXERCISE PROGRAM** is designed to help you in your transition from running with a rear foot strike pattern to a non-rear foot strike pattern. By performing the stretches, drills and strengthening exercises below, you will decrease the forces your body must absorb. When runners change their running form, foot and lower leg soreness are a common side effect. In many cases, the soreness can be alleviated with proper stretching, controlled movements and gradual exercise progressions like the one below.

CALF STRETCHING

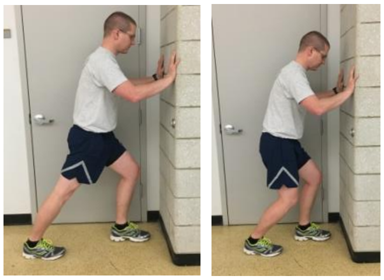

Perform 3 x 30 sec, 1-2x/day. Keep heel on ground. Perform with straight & bent knee.

CALF FOAM ROLLING

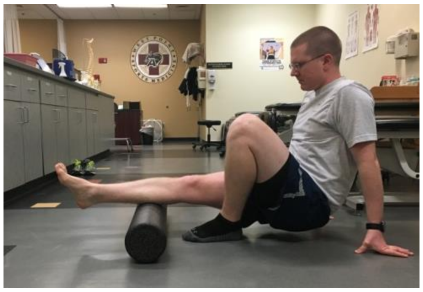

Perform 2-3 min/day. Roll up/down calf muscles with weight as tolerated.

PLANTAR FASCIAL STRETCH

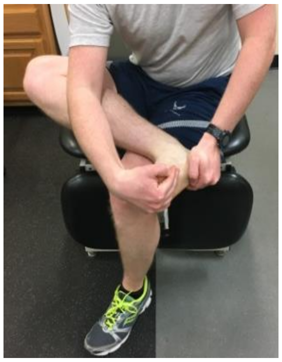

Perform 3 x 30 sec, 1-2x/day. Stabilize inside of heel, pull ball on foot/toes to shin.

WEIGHT SHIFTING

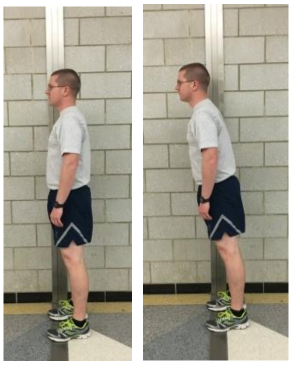

Perform 10 reps, 2-3x/week. Shift body weight from heels to forefeet. May also close eyes and/or perform barefoot.

FOOT TAPPING

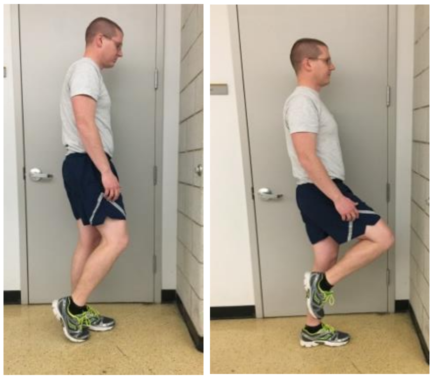

Perform 10 reps, 2-3x/week. Rest toes on ground and use hamstring to pull foot up the length of opposite leg. Allow foot to fall down with gravity.

MARCHING ON FOREFEET

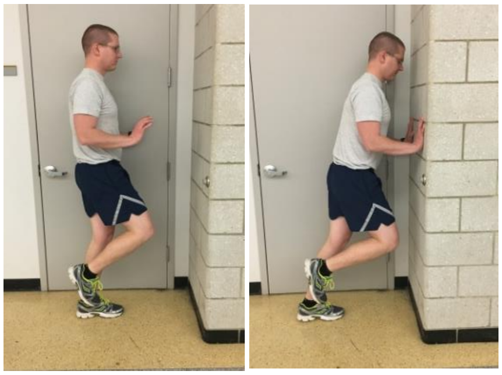

Perform 10 reps, 2-3x/week. Fall into the wall, shifting weight onto forefoot. Once leaned forward, tap heel on ground. Then alternate feet (marching) with forefoot landing first followed by heel tap.

TOE YOGA

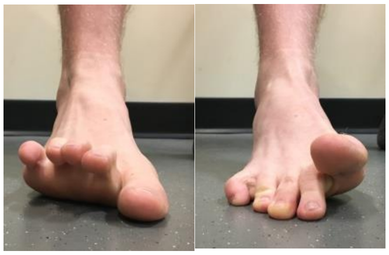

Perform 3 x 30 sec, 1-2x/week. Alternate lifting big toe/little toes independently of each other off of the ground. May also perform inside shoes.

KNEE TO CHEST BRIDGE

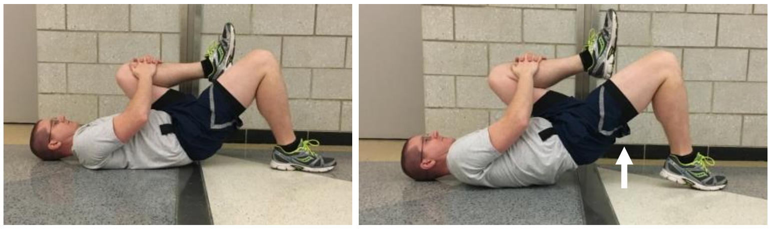

Perform 10 reps, 2-3x/week. Pull and hold knee to chest. Using opposite leg, lift hips into a bridge position holding for 5-10 seconds. Lift heel off the ground as shown.

Homemade ice pack: Combine 2/3 water with 1/3 rubbing alcohol in a Ziploc bag, freeze. Use for 15 minutes as needed
