# Supplementary material for: Wearable Technology May Assist in Retraining Foot Strike Patterns in Previously Injured Military Service Members: A Prospective Case Series
Source: Front Sports Act Living. 2021 Feb 26;3:630937. doi: 10.3389/fspor.2021.630937 (PMC7952986; doi:10.3389/fspor.2021.630937)
Supplement: Supplementary file 4 [file Data_Sheet_4.PDF]

Supplement 4. Repeated measures analysis of variance pairwise comparison with Holm correction for participants who transitioned to a NRFS running pattern with a history of unilateral knee injury or surgery (n = 9[#]).

Variable		Initial data collection (T1)	Post-intervention data collection[†] (T2)	Follow-up data collection[ϕ] (T3)	Notes
NPRS		0.00 (0.00, 0.00)	1.44 (-0.37, 3.25)	0.33 (-0.22, 0.71)	T1 – T2: $p = 0.31$<br>T2 – T3: $p = 0.39$<br>T1 – T3: $p = 0.39$
PSFS		45.89 (34.18, 57.60)	49.89 (41.86, 57.92)	54.33 (47.19, 61.47)	T1 – T2: $p = 0.25$<br>T2 – T3: $p = 0.06$<br>T1 – T3: $p = 0.08$
SANE		84.44 (74.19, 94.69)	82.11 (68.60, 95.62)	87.17 (74.34, 99.99)	T1 – T2: $p = 1.00$<br>T2 – T3: $p = 0.23$<br>T1 – T3: $p = 1.00$
LEFS		72.78 (67.85, 77.71)	73.22 (68.02, 78.42)	74.22 (67.85, 80.59)	T1 – T2: $p = 1.00$<br>T2 – T3: $p = 1.00$<br>T1 – T3: $p = 1.00$
Average vertical loading rate (BW/sec)	Involved	57.75 (45.84, 69.66)	39.93 (34.03, 45.83)	40.81 (32.02, 49.60)	T1 – T2: $p = 0.04$*<br>T2 – T3: $p = 0.78$<br>T1 – T3: $p = 0.04$*
Average vertical loading rate (BW/sec)	Uninvolved	58.74 (46.16, 71.32)	40.56 (34.75, 46.37)	43.09 (38.54, 47.64)	T1 – T2: $p = 0.07$<br>T2 – T3: $p = 0.10$<br>T1 – T3: $p = 0.07$
Cadence (steps/min)	Mean, Involved and Uninvolved	166.27 (159.93, 172.61)	173.98 (168.70, 179.26)	172.39 (166.31, 178.47)	T1 – T2: $p = 0.08$<br>T2 – T3: $p = 0.45$<br>T1 – T3: $p = 0.01$*
Peak vertical GRF (BW)	Involved	2.34 (2.16, 2.52)	2.42 (2.24, 2.60)	2.43 (2.25, 2.61)	T1 – T2: $p = 0.68$<br>T2 – T3: $p = 0.68$<br>T1 – T3: $p = 0.68$
Peak vertical GRF (BW)	Uninvolved	2.36 (2.14, 2.58)	2.39 (2.19, 2.59)	2.45 (2.20, 2.70)	T1 – T2: $p = 0.68$<br>T2 – T3: $p = 0.63$<br>T1 – T3: $p = 0.68$
Stance Time (sec)	Involved	0.26 (0.24, 0.28)	0.24 (0.22, 0.26)	0.24 (0.22, 0.26)	T1 – T2: $p < 0.001$*<br>T2 – T3: $p = 0.64$<br>T1 – T3: $p < 0.001$*
Stance Time (sec)	Uninvolved	0.26 (0.24, 0.28)	0.24 (0.22, 0.26)	0.24 (0.22, 0.26)	T1 – T2: $p = 0.002$*<br>T2 – T3: $p = 0.37$<br>T1 – T3: $p = 0.003$*

NRFS – Non-rearfoot strike; NPRS – Numerical pain rating scale; PSFS – Patient specific functional scale; SANE – Single assessment numerical evaluation; LEFS – Lower extremity functional scale; GRF – Ground reaction force; BW – Body weights.

These data are reported as mean and 95% confidence interval for the involved and uninvolved lower extremity, unless otherwise noted.

[#] One participant did not transition to a NRFS running pattern and was excluded from these data.

[†] Mean days from initial to post-intervention data collection: 52.09 ± 8.65 days.

[ϕ] Mean days from post-intervention to follow-up data collection: 34.82 ± 6.27 days.

* Denotes significance ($p < 0.05$).
